# A Mouse Model of Latent Tuberculosis Infection to Study Intervention Strategies to Prevent Reactivation

**DOI:** 10.1371/journal.pone.0158849

**Published:** 2016-07-08

**Authors:** Andreas Kupz, Ulrike Zedler, Manuela Stäber, Stefan H. E. Kaufmann

**Affiliations:** 1 Department of Immunology, Max Planck Institute for Infection Biology, Berlin, Germany; 2 Centre for Biosecurity and Tropical Infectious Diseases, Australian Institute of Tropical Health and Medicine, James Cook University, Cairns, QLD, Australia; IPBS, FRANCE

## Abstract

Infection with *Mycobacterium tuberculosis* (*Mtb*) is the leading cause of death in human immunodeficiency virus (HIV)^+^ individuals, particularly in Sub-Saharan Africa. Management of this deadly co-infection is a significant global health challenge that is exacerbated by the lack of efficient vaccines against both *Mtb* and HIV, as well as the lack of reliable and robust animal models for *Mtb*/HIV co-infection. Here we describe a tractable and reproducible mouse model to study the reactivation dynamics of latent *Mtb* infection following the loss of CD4^+^ T cells as it occurs in HIV-co-infected individuals. Whereas intradermally (i.d.) infected C57BL/6 mice contained *Mtb* within the local draining lymph nodes, depletion of CD4^+^ cells led to progressive systemic spread of the bacteria and induction of lung pathology. To interrogate whether reactivation of *Mtb* after CD4^+^ T cell depletion can be reversed, we employed interleukin (IL)-2/anti-IL-2 complex-mediated cell boost approaches. Although populations of non-CD4 lymphocytes, such as CD8^+^ memory T cells, natural killer (NK) cells and double-negative (DN) T cells significantly expanded after IL-2/anti-IL-2 complex treatment, progressive development of bacteremia and pathologic lung alterations could not be prevented. These data suggest that the failure to reverse *Mtb* reactivation is likely not due to anergy of the expanded cell subsets and rather indicates a limited potential for IL-2-complex-based therapies in the management of *Mtb*/HIV co-infection.

## Introduction

Infections with *Mtb*, the causative agent of tuberculosis (TB), are the major cause of death in human immunodeficiency virus (HIV)^+^ individuals, accounting for approximately 25% of all HIV-related fatalities [1]. Together, TB and HIV/AIDS account for more than 2 million deaths per year [1, 2] and HIV^+^ individuals are 30 times more likely to develop active TB than HIV^−^ individuals [2]. Both pathogens profoundly impact on the human immune system and the gradual decline of CD4^+^ T cells, the hallmark of HIV infection, is believed to be a major contributing factor in the progression to active TB disease and premature death [3]. CD4^+^ T cells are not only prime producers of interferon gamma (IFN-γ), a critical cytokine for TB control, but also play essential roles in the maturation of the humoral immune response and the structural integrity of *Mtb*-containing solid granulomas [4]. Particularly the HIV-induced preferential destruction of mucosal effector memory CD4^+^ T cells [5] and CD1-restricted mycobacterial glycolipid-reactive T cells [6], as well as the imbalance between Th17 and regulatory T cell (Treg) populations [7] apparently favour *Mtb* reactivation. Although anti-retroviral therapy (ART) largely restores CD4^+^ T cell numbers, the increased risk for reactivation of latent TB infection (LTBI) is only partially diminished [8]. In order to develop intervention strategies against *Mtb* that are also effective in HIV^+^ individuals, it is therefore useful to identify CD4-independent mechanisms which contribute to control of *Mtb* infection.

We have recently described a critical role for non-cognate production of IFN-γ by NK cells, memory CD8^+^ T cells and DN T cells in TB [9]. Rapid secretion of IFN-γ by these lymphocyte populations in TB required the sensing of ESAT-6-mediated cytosolic contact via NLRP3 inflammasomes within CD11c^+^ cells and the subsequent secretion of bioactive interleukin (IL)-18. These results not only delineate a mechanistic framework for IFN-γ production by IL-18 responsive cell types after *Mtb* infection, but also indicate an inherent capacity of non-CD4 immune cells to contribute to protective immunity. In order to potentially harness non-CD4 immune cell subsets in settings where adaptive immune responses are absent or impaired, such as *Mtb*/HIV co-infection, it would be desirable to not only boost their numbers and differentiation capacity but also to induce IFN-γ secretion.

The development, maturation and maintenance of NK cells, CD8^+^ memory T cells and most DN T cells relies on IL-15 and IL-2 [10–15]. The activity of these two cytokines is mediated through trans-presentation, a mechanism by which the cytokine is presented to the cytokine receptor complex beta and common γ chains in the context of cell-bound high-affinity alpha (α) chains of the cytokine receptor [16, 17]. Both IL-2/anti-IL-2 and IL-15/IL-15RαFc complexes have been shown to boost NK cell and memory CD8^+^ T cell numbers in mice and to enhance their cytolytic capacity against viral-infected and cancer cells [16, 18–20]. Short-term exposure of naïve mice with IL-2 complexes containing the anti-IL-2 mAb clone S4B6 has also been shown to enhance resistance and immunity against subsequent *Listeria monocytogenes* infection [21]. Additionally, IL-2 complexes containing the anti-IL-2 mAb clone JES6-1A12 have been studied for their beneficial role in Treg expansion and activation in models of autoimmunity, infection and cancer [22]. However, whether IL-2/anti-IL-2 cytokine complex-mediated cell expansion can be harnessed as an effective measure to boost immunity against active TB, or to prevent TB reactivation after HIV co-infection, is not known.

A critical limitation for studies on *Mtb*/HIV co-infection is the lack of appropriate animal models. Although several mouse and non-human primate models for TB have been developed [23], these models are largely unsuitable to study HIV co-infection due to HIV’s restriction to human cells. Simian immunodeficiency virus (SIV) infections in macaques mimic human HIV infection, but for numerous reasons including ethical, financial and logistical ones this model has substantial limitations. Similarly, the use of immunocompromised mice engrafted with human hematopoietic progenitor cells (‘humanized mice’) [24] or infections of conventional mice with ‘EcoHIV’, a modified HIV strain which infects mice [25], have only limited potential as reliable, affordable and tractable HIV/*Mtb* co-infection animal models.

Our group has previously developed a murine TB ear dermis infection model that reflects several aspects of human TB [26, 27]. In this model, *Mtb* only spreads to the spleen and lung in mice lacking inducible nitric oxide synthase (iNOS) and these mice also develop human-like lung granulomas [26]. In contrast, fully immunocompetent mice contain *Mtb* within the draining lymph node (LN) of the ear. We hypothesized that the transition from containment to systemic spread depending on the immunological status of the host should allow reactivation of latent TB in this model through depletion of CD4^+^ T cells. Recently it was proposed that TB has characteristic features of lymphatic diseases with a pulmonary portal and that pulmonary pathology primarily serves *Mtb* transmission [28]. In this context, using a latent lymphatic *Mtb* infection model presents an opportunity to study *Mtb* reactivation from a lymphocentric perspective. The present study examines a modified version of the murine ear dermis *Mtb* infection model in conjunction with IL-2/anti-IL-2 complex treatment as a model for host-directed immunotherapy to specifically boost and activate NK cells and CD8^+^ memory T cells during progression to active TB as a consequence of CD4^+^ T cell deficiency.

## Results

### Depletion of CD4^+^ T cells reactivates chronic lymphatic *Mtb* infection

**Fig 1:**
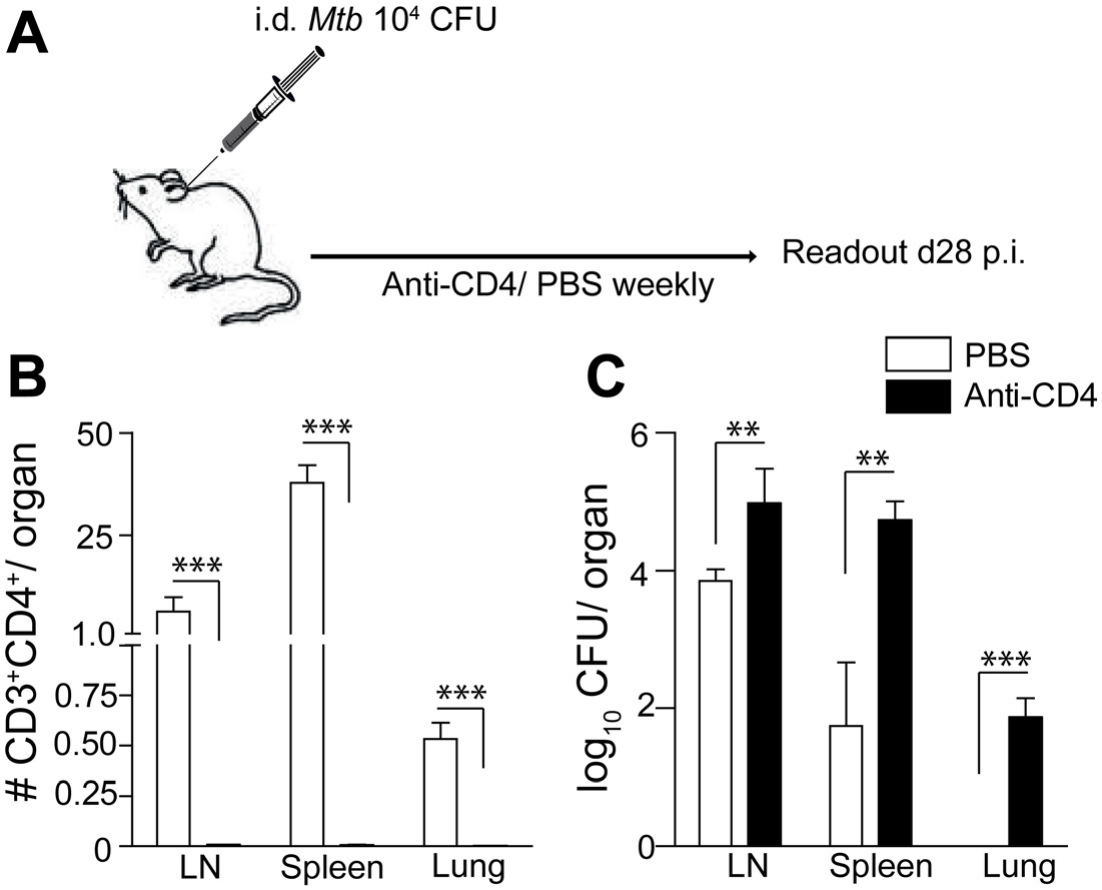
Depletion of CD4^+^ T cells reactivates chronic lymphatic *Mtb* infection. Naïve B6 mice were infected i.d. with 1×10^4^ *Mtb* H37Rv. At weekly intervals, mice received intraperitoneally (i.p.) a mAb against mouse CD4 (GK1.5) or PBS (A). On day 28 p.i mice were sacrificed and ear-draining LNs, spleen and lung were assessed for numbers of CD4^+^ T cells by FACS (B) and numbers of viable bacteria (C). Results are presented as pooled data means ± SEM from one representative experiment (n = 4 mice per group). Statistical analyses: Mann-Whitney *U*-test per organ; significant differences are indicated by asterisks: ** p<0.01; *** p<0.001.

To study progression from LTBI to active TB as a consequence of CD4^+^ T cell deficiency, as it occurs in HIV co-infection, we first adapted the murine ear dermis *Mtb* infection model [26, 27]. To this end, WT B6 mice were infected with 1 × 10^4^ *Mtb* H37Rv in the ear dermis and thereafter treated weekly with either anti-CD4 monoclonal antibody (mAb) (GK1.5) or PBS (Fig 1A). Twenty eight days post-infection (p.i), the bacterial burden in ear-draining LNs, spleen and lung was assessed. PBS-treated animals almost exclusively contained *Mtb* within the draining LNs of the infected ear (Fig 1C). In some animals few bacteria were detected in the spleen but never in the lung (Fig 1C). In contrast, in recipients of anti-CD4 mAb, *Mtb* not only multiplied significantly within the LNs but also quickly exited the ear-draining LNs and spread to spleen and lung in all animals (Fig 1C). Given that CD4^+^ T cells were efficiently depleted in spleen, lung and LN in anti-CD4 mAb-treated animals (Fig 1B), we reasoned that this model resembles progression from LTBI to active TB in human HIV/*Mtb* co-infection.

### Non-CD4 immune cells can be expanded in WT and CD4-deficient mice

**Fig 2:**
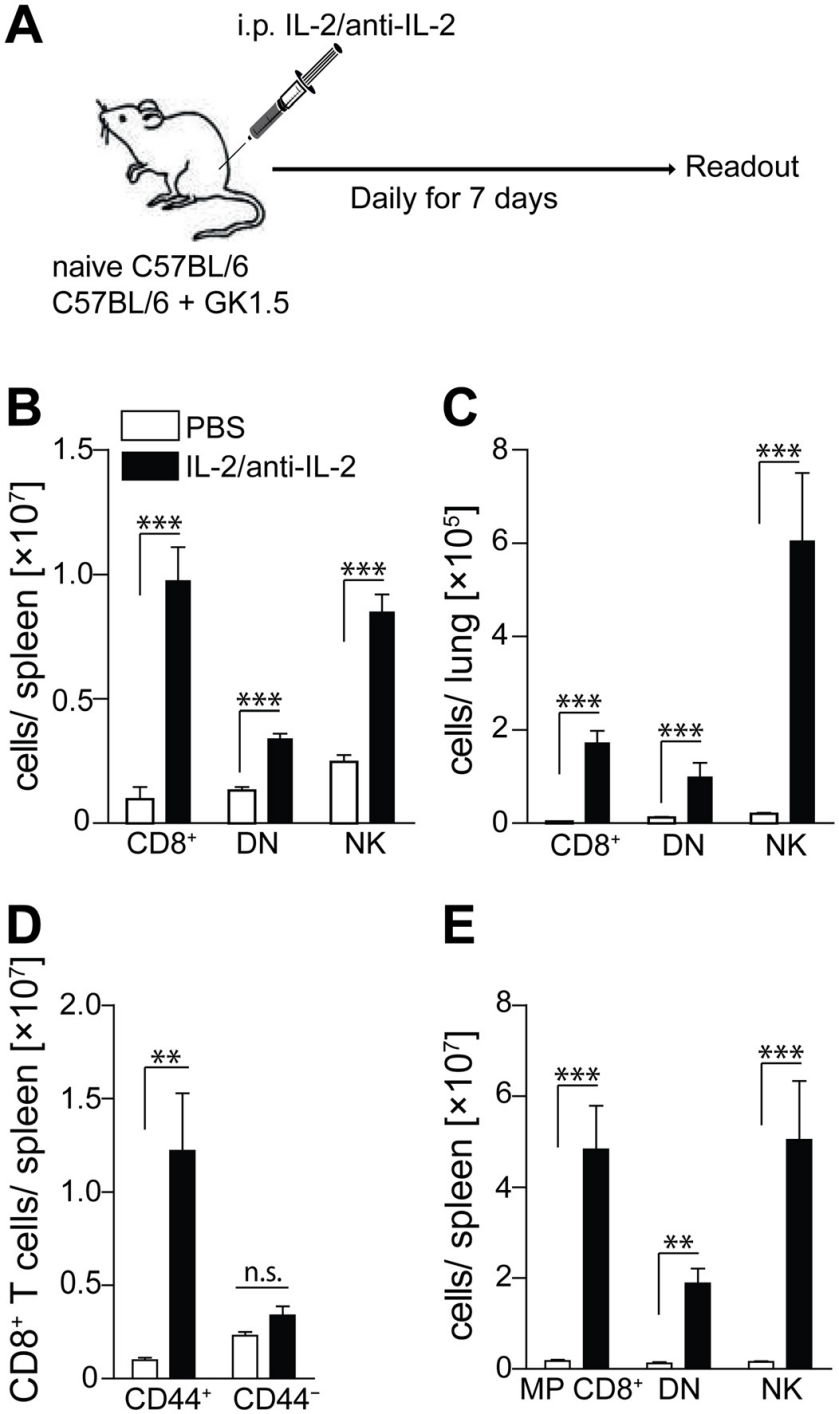
Non-CD4 immune cells can be numerically expanded by treatment with IL-2/anti-IL-2 complexes. Naïve B6 mice (A, D) and anti-CD4-treated B6 mice (C, E) were treated i.p. with IL-2/anti-IL-2 complexes on seven consecutive days (A). One day after the last administration, mice were sacrificed and numbers of CD3^+^CD8^+^, CD3^+^CD8^+^CD44^+^, CD3^+^CD8^+^CD44^+^, CD3^+^CD4^−^CD8^−^ (DN) and CD3^−^NK1.1^+^ cells in spleen (B, D, E) and lung (C) were assessed by FACS. Results are presented as pooled data means ± SEM from two pooled independent experiments (n = 8 mice per group). Statistical analyses: One-way ANOVA followed by Bonferroni multiple comparison test for non-parametric samples; significant differences are indicated by asterisks: ** p<0.01; *** p<0.001; n.s. not significant.

Next we assessed whether non-CD4 lymphocyte subsets that have been implicated in early control of *Mtb* [9], can be targeted with IL-2/anti-IL-2 complexes to compensate for the loss of CD4^+^ T cells [20, 22]. To this end, naïve WT mice and CD4-depleted mice were treated with IL-2/anti-IL-2 complexes or PBS and *Mtb*-unrelated memory CD8^+^ T cells, NK cells and DN T cells were enumerated (Fig 2A). In line with published data, the IL-2/anti-IL-2 complex containing the anti-IL-2 mAb clone S4B6 but not the mAb clone JES6-5H4 for seven consecutive days [22] caused a significant (>30 fold) expansion of CD8^+^ T cells, DN T cells and NK cells in spleen, lung and LNs of naïve B6 mice (Fig 2B and 2C and data not shown). Within the CD8^+^ T cell compartment, IL-2/anti-IL-2-complex-treatment only expanded CD44^+^ but not CD44^−^ cells, indicating its selective beneficial effect on memory CD8^+^ T cells (Fig 2D). Moreover, when B6 mice were additionally treated with anti-CD4 mAb prior to IL-2/anti-IL-2 complex, the numerical expansion of memory CD8^+^ T cells, DN T cells and NK cells was even more dramatic (Fig 2E). Collectively, these results demonstrate that non-CD4 immune cell populations, which contribute to early protection against TB [9], can be significantly expanded using IL-2/anti-IL-2 complex in both WT and CD4-deficient mice.

### Numerical expansion of non-CD4 immune cells fails to reverse TB reactivation

**Fig 3:**
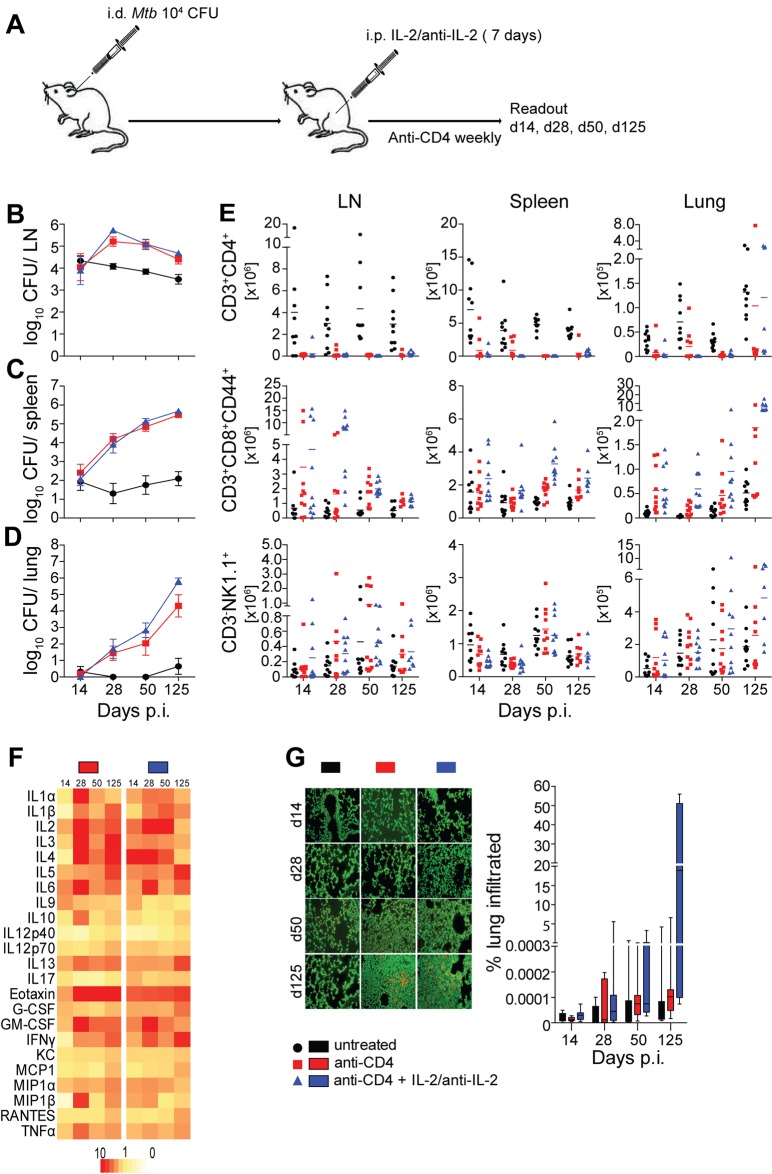
Expansion of non-CD4 immune cell populations fails to reverse reactivation of lymphatic LTBI. Naïve B6 mice were infected i.d. with 1×10^4^ *Mtb* H37Rv. In weekly intervals mice received a depleting mAb against mouse CD4 (GK1.5) or PBS i.p. One group of mice received IL-2/anti-IL-2 complexes i.p. on seven consecutive days (A). On days 14, 28, 50 and 125 p.i. mice were sacrificed and ear draining LNs, spleen and lung were assessed for numbers of viable bacteria (B–D) and numbers of CD3^+^CD4^+^, CD3^+^CD8^+^CD44^+^ and CD3^−^NK1.1^+^ cells (E). Serum concentrations of cytokines (F) and lung pathology (G) were determined. Results are presented as pooled data means ± SEM (B–D, G), individual data points (E), fold upregulation relative to untreated controls (F) and representative images (G) from three pooled independent experiments (n = 7–10 mice per group).

In order to assess the impact of IL-2/anti-IL-2 complex-mediated expansion of non-CD4 immune cell populations in the murine ear dermis infection model, we combined the anti-CD4-mediated reactivation of *Mtb* (Fig 1) with the expansion of non-CD4 immune cell populations (Fig 2). To this end, B6 mice were i.d. infected with 1 × 10^4^ *Mtb* H37Rv. Subsequently two groups of mice were treated weekly with anti-CD4 mAb and one of these groups additionally with IL2/anti-IL-2 complexes for seven consecutive days (Fig 3A). Recipients of anti-CD4 mAb only showed a progressive increase in bacterial numbers in LNs, spleen and lung over time (Fig 3B–3D). Additional IL-2/anti-IL-2 complex treatment did not decrease numbers of viable bacteria in LNs, spleen or lung (Fig 3B–3D). These data indicate that the sole expansion of non-CD4 T cell populations is insufficient to restrict bacterial growth after reactivation of LTBI.

Additionally, we performed a comprehensive cytokine and lung histology analysis over time. Although cells expanded as expected (Fig 3E), IL2/anti-IL-2 treatment increased lung pathology over time (Fig 3G). Furthermore, although the IL-2/anti-IL-2 treatment appeared to reduce the upregulation of pro-inflammatory cytokines only within the first 28 days, an obvious and consistent shift within the cytokine profile was not observed long-term (Fig 3F). Taken together these results indicate that IL-2/anti-IL-2-mediated expansion of non-CD4 lymphocyte populations fails to prevent reactivation of LTBI in this model. The overall exacerbation of bacterial burden and lung pathology over time even indicates that significant detrimental adverse events were induced by these treatments.

### Shorter IL-2/anti-IL-2 treatment reduces immune pathology but not bacterial burden

**Fig 4:**
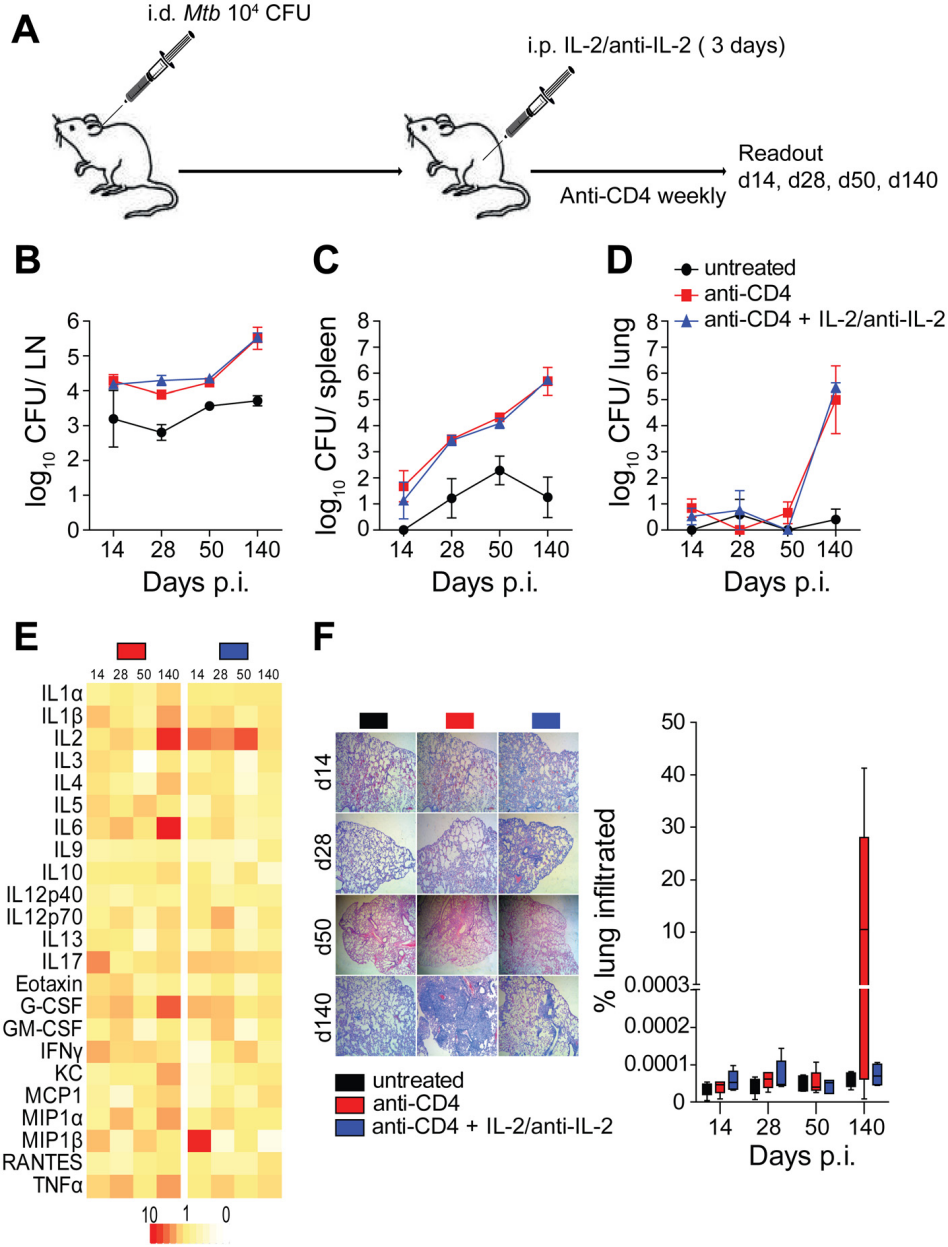
T cell anergy does not explain failure to reverse *Mtb* reactivation. Naïve B6 mice were infected i.d. with 1×10^4^ *Mtb* H37Rv. In weekly intervals, mice received a depleting mAb against mouse CD4 (GK1.5) or PBS i.p. One group of mice received IL-2/anti-IL-2 complexes i.p. on three consecutive days (A). On days 14, 28, 50 and 125 p.i., mice were sacrificed and ear-draining LNs, spleen and lung were assessed for numbers of viable *Mtb* (B–D). Additionally, serum cytokine concentrations (E) and lung pathology (F) were determined. Results are presented as pooled data means ± SEM (B–D, F), fold upregulation relative to untreated controls (e) and representative images (f) from two pooled independent experiments (n = 7–10 mice per group).

Although initial studies described a maximum cell expansion after six to seven administrations of IL-2/anti-IL-2 complex [22], it was later found that such prolonged exposure to IL-2/anti-IL-2 complexes can lead to T cell anergy in mice [21]. In these latter studies only formulations with three but not five IL-2/anti-IL2 administrations protected mice from subsequent *Listeria monocytogenes* and vaccinia virus infection [21]. To explore whether the failure of IL-2/anti-IL-2 treatment to reverse lymphatic *Mtb* reactivation in our model was due to T cell anergy, we repeated the experiment described in Fig 3 with three instead of seven consecutive administrations of IL-2/anti-IL-2 complex (Fig 4A).

Consistent with our previous observations, mice treated with anti-CD4 mAb displayed elevated numbers of *Mtb* in LNs over time and progressively increasing numbers were detected in spleen and lung (Fig 4B–4D). However, no reduction of *Mtb* numbers was observed when mice were additionally treated with three doses of IL-2/anti-IL-2 complex (Fig 4B–4D). In contrast to the longer IL-2/anti-IL-2 treatment, three doses significantly reduced abundance of inflammatory cytokines, lung pathology and general inflammation at later time points (Fig 4E and 4F). Although severity of lung pathology was greatly reduced by IL-2/anti-IL-2 complex treatment, *Mtb* numbers in the lung were almost indistinguishable between anti-CD4 treated animals and anti-CD4 + IL-2/anti-IL-2-treated mice at day 140 p.i. These results indicate that despite the failure of IL-2/anti-IL-2 complex treatment to prevent TB reactivation, lung pathology was ameliorated by this treatment regimen. Hence further investigations into the effects of IL-2/anti-IL-2 complex-mediated expansion of non-CD4 immune cells are warranted.

## Discussion

Here we describe a tractable mouse model to study latent lymphatic *Mtb* infection, the progression to active TB after loss of CD4^+^ T cells and the impact of preventive expansion of non-CD4 lymphocytes by host-directed therapy. More specifically, we show that i.d. infection of B6 mice with *Mtb* resulted in LTBI restricted to ear-draining LNs. Treatment of latently infected mice with anti-CD4 mAb caused TB reactivation and systemic spread of *Mtb* to spleen and lung, mimicking the rapid progression from LTBI to TB in HIV^+^ individuals. Additionally, we report that prophylactic host-directed therapy with IL-2/anti-IL-2 complexes to expand non-CD4 lymphocytes participating in protection against TB, fails to prevent TB reactivation in this model. Although our results indicate limited potential of IL-2/anti-IL-2-mediated cell expansion as adjunctive host-directed therapy in *Mtb*/HIV co-infected individuals, the mouse model described can be further exploited to study other aspects of *Mtb* reactivation and other treatment regimens.

During the development of ART, recombinant IL-2 has been trialed as adjunctive AIDS treatment by boosting CD4^+^ T cell numbers during ART. Indeed these approaches caused substantial and sustained increases in CD4^+^ T cell numbers yet; no clinically significant benefits of IL-2 treatment over ART alone were observed [29–31]. IL-2 treatment has been more successfully used for treatment of persistent co-infections with drug-resistant mycobacteria in HIV^+^ individuals [32]. These studies aimed at numerical expansion of CD4^+^ T cell numbers to ameliorate infections with various opportunistic bacterial and parasitic pathogens in AIDS patients. Given the better understanding of the role of non-CD4^+^ immune cells in early control of severe bacterial infections, including *Mtb* and non-typhoidal *Salmonella* [9, 33, 34], we assessed beneficial effects of boosting these cell types with IL-2/anti-IL-2 complexes. The apparent failure of this approach in our newly established mouse model of progression from LTBI to active TB could be due to the following non-mutually exclusive explanations: (i) transient expansion of non-CD4 cell populations after IL-2/anti-IL-2 treatment; (ii) requirement of prophylactic rather than preventive expansion of cell populations; (iii) activation of a pro-inflammatory cytokine ‘storm’ that supports rather than limits initial *Mtb* replication; and (iv) toxic side effects of high doses of IL-2, such as vascular leakage syndrome [35], promoting *Mtb* spread. It is also unclear whether the IL-2/anti-IL-2 complexes remained stable and how much free IL-2 was administered. This obstacle could be overcome by using a stable chimeric protein of IL-2 and the anti-IL-2 clone S4B6 [36]. The hyper inflammatory response caused by IL-2/anti-IL-2 treatment in our model could also resemble aspects of the immune reconstitution inflammatory syndrome (IRIS) that is frequently seen in *Mtb*-infected AIDS patients on ART after recovery of the immune system begins [37]. Furthermore, *in vivo* administration of anti-CD4 mAb likely also affects other *Mtb*-containing CD4^+^ cell types such as dendritic cells and macrophages. Depletion of these cell types may also impact the containment of LTBI in our model and may mask any beneficial effects of IL-2/anti-IL-2 treatment. All of these issues require further investigations and more refined models.

IL-2 complexes have been used successfully in several other models. For example, Kanimura and colleagues have trialed IL-2/anti-IL-2 complex treatment in a mouse model of B16 melanoma [38], and Tomala and colleagues in a mouse model of BCL1 leukemia [39] as cancer immunotherapy. In these models, IL-2/anti-IL-2 complexes administered early after tumor inoculation significantly increased the survival rate of mice [35]. Similarly, in a mouse model of gamma-herpesvirus infection, IL-2 complex-mediated expansion of CD8^+^ T and NK cell populations significantly reduced viral load in a perforin- and granzyme-dependent, IFN-γ-independent manner [40]. To our knowledge, no data are available yet on any clinical use of IL-2/anti-IL-2 complexes in humans.

IL-15/IL-15Rα-Fc complexes have been shown to significantly expand populations of memory CD8^+^ T cells, NK cells and DN T cells. In contrast to IL-2, IL-15 operates as a membrane-bound complex in conjunction with the IL-15Rα-chain. This process, termed trans-presentation, allows more direct and more fine-tuned delivery of the cytokine signal to the recipient cell [35], a relevant feature for clinical application. Additionally, IL-15/IL-15Rα-Fc complexes do not induce Treg cells, as some IL-2/anti-IL-2 complexes do, and could therefore have more potent effects. Whether IL-15/IL-15Rα-Fc complexes would perform better than IL-2/anti-IL-2 complexes in our model of *Mtb* reactivation remains to be investigated.

In summary, our results provide a tractable and reproducible mouse model to study the dynamics of progression from lymphatic LTBI to active TB following the loss of CD4^+^ T cells. Furthermore, our findings suggest only limited potential of IL-2/anti-IL-2 complexes as adjunctive therapy of *Mtb*/HIV co-infection. Finally, these results underpin the importance of appropriate models to mimic *Mtb*/HIV co-infection which allow broad-scale testing of host directed therapies.

## Materials and Methods

### Ethics Statement

All experiments were conducted in accordance with requirements of and approval by the State Office for Health and Social Services (*Landesamt für Gesundheit und Soziales*), Berlin, Germany (G0031/13, T 0087/13).

### Mice

C57BL/6 mice were purchased from Charles River, Sulzfeld, Germany and maintained in the animal facilities of the Max Planck Institute for Infection Biology, Berlin, Germany. For infection experiments, all mice were sex- and age-matched, and kept in-house in a biosafety level 3 facility under specific pathogen free (SPF) conditions. Mice were sacrificed by cervical dislocation.

### Bacteria

*Mtb* H37Rv was grown in Middlebrook 7H9 broth (BD Biosciences) supplemented with 0.2% glycerol, 0.05% Tween 80 and 10% ADC enrichment (BD Biosciences) Mid-logarithmic cultures were harvested, washed in PBS and stored at –80°C.

### Infections and enumeration of bacteria

For i.d. infections, mice were anesthetized via i.p. injections of Rompun (5 mg/kg; Bayer) and ketamin (50 mg/kg) and were subsequently infected with 1×10^4^ cfu *Mtb* H37Rv in the ear dermis in a volume of 50 μl. At designated time points, serial dilutions of tissue homogenates were plated onto Middlebrook 7H11 agar supplemented with 10% OADC Enrichment (BD Biosciences) and ampicillin (25 μg/ml). Cfu were determined after 3–4 weeks incubation at 37°C.

### mAb-mediated depletion of CD4^+^ T cells and assessment of depletion

CD4^+^ T cells were depleted from B6 mice by weekly i.p. injections of 200 μg mAb against CD4 (clone GK1.5). mAb were produced in-house from hybridoma cell lines.

### IL-2/anti-IL-2 complex-mediated cell expansion

IL-2/anti-IL-2 complexes were prepared as previously described [22, 41]. Briefly, 1.5 μg of recombinant mouse IL-2 (eBiosciences) and 10 μg of anti-IL-2 mAb (clone S4B6, produced in house) were mixed, incubated at 37°C for 30 min, and administered i.p. in a volume of 200 μl for up to seven consecutive days.

### Isolation of lung leukocytes

Lungs were perfused with PBS, extracted, mechanically disrupted and digested via incubation for 30 min with RPMI 1640 medium supplemented with glutamine, Na-pyruvate, 2-ME, penicillin, streptomycin, 10% heat-inactivated FCS, collagenase D (Roche) and collagenase type VIII (Sigma-Aldrich). Subsequently, red blood cell depleted, single-cell suspensions were prepared as described elsewhere.

### Flow cytometry

To assess expression of surface antigens, viable, red blood cell-depleted single splenocytes were stained with mAb (all from BD Pharmingen) against CD4 (GK1.5), CD8α (53–6.7), CD3 (145-2C11), CD44 (1M7) and NK1.1 (PK136) as described elsewhere. After washing the cells, samples were analyzed using a FACSCantoII or LSRII analyzers (BD Biosciences, CA). Propidium iodide (2 μg/ ml) was added to exclude dead cells.

### Histology

Lung lobes were collected aseptically, fixed overnight with 4% w/v paraformaldehyde and embedded in paraffin. Two-μm tissues sections were stained with Giemsa or TB Flour according to standard protocols as described elsewhere [42].

### Multiplex

Blood for serum analysis was taken post mortem from the aorta abdominalis and collected in serum separator tubes (BD), left for 30 min at room temperature, followed by centrifugation at 12,000 × *g* for 3 min. Sera were stored at –20°C until analysis. Measurements were performed using a multiplex bead-based immunoassay kits (Bio-Rad) according to manufacturer’s instructions. Samples were acquired on a Bio-Rad instrument.

### Data and statistical analysis

Flow cytometry data was analyzed using FlowJo software (Treestar, CA) and statistical analysis was performed using GraphPad Prism Version 5.04, GraphPad software, San Diego, CA as indicated in individual figure legends.
